# Acquired small cell lung cancer resistance to Chk1 inhibitors involves Wee1 up‐regulation

**DOI:** 10.1002/1878-0261.12882

**Published:** 2021-01-26

**Authors:** Xiaoliang Zhao, In‐Kyu Kim, Bhaskar Kallakury, Joeffrey J. Chahine, Eiji Iwama, Mariaelena Pierobon, Emanuel Petricoin, Justine N. McCutcheon, Yu‐Wen Zhang, Shigeki Umemura, Vincent Chen, Changli Wang, Giuseppe Giaccone

**Affiliations:** ^1^ Lombardi Comprehensive Cancer Center Georgetown University Washington DC USA; ^2^ Department of Lung Cancer Tianjin Key Laboratory of Cancer Prevention and Therapy National Clinical Research Center for Cancer Tianjin Medical University Cancer Institute and Hospital China; ^3^ Department of Surgery Open NBI Convergence Technology Research Laboratory Yonsei Cancer Center Severance Hospital Yonsei University College of Medicine Seoul South Korea; ^4^ George Mason University Manassas VA USA

**Keywords:** acquired resistance, cell cycle, Chk1 inhibitor, Wee1

## Abstract

Platinum‐based chemotherapy has been the cornerstone treatment for small cell lung cancer (SCLC) for decades, but no major progress has been made in the past 20 years with regard to overcoming chemoresistance. As the cell cycle checkpoint kinase 1 (Chk1) plays a key role in DNA damage response to chemotherapeutic drugs, we explored the mechanisms of acquired drug resistance to the Chk1 inhibitor prexasertib in SCLC. We established prexasertib resistance in two SCLC cell lines and found that DNA copy number, messengerRNA (mRNA) and protein levels of the cell cycle regulator Wee1 significantly correlate with the level of acquired resistance. Wee1 small interfering RNA (siRNA) or Wee1 inhibitor reversed prexasertib resistance, whereas Wee1 transfection induced prexasertib resistance in parental cells. Reverse phase protein microarray identified up‐regulated proteins in the resistant cell lines that are involved in apoptosis, cell proliferation and cell cycle. Down‐regulation of CDK1 and CDC25C kinases promoted acquired resistance in parental cells, whereas down‐regulation of p38MAPK reversed the resistance. High Wee1 expression was significantly correlated with better prognosis of resected SCLC patients. Our results indicate that Wee1 overexpression plays an important role in acquired resistance to Chk1 inhibition. We also show that bypass activation of the p38MAPK signaling pathway may contribute to acquired resistance to Chk1 inhibition. The combination of Chk1 and Wee1 inhibitors may provide a new therapeutic strategy for the treatment of SCLC.

## Graphical abstract

Wee1 up‐regulation is a major and novel mechanism of acquired resistance to Chk1 inhibitors in SCLC. Wee1 expression levels are correlated with resistance levels. Combination of Chk1 and Wee1 inhibitors may overcome this resistance. Combination studies of Chk1 inhibitors such as prexasertib and other agents may lead to synergistic interactions. Combinations with immune check‐point inhibitors might also be of particular interest.

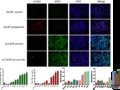

**Abbreviations**
ATM: ataxia telangiectasia mutated
ATR: ATM and Rad‐3‐related
CDK: cyclin‐dependent kinase
Chk1: checkpoint kinase 1
CI: combination index
CNV: copy number variation
DDR: DNA Damage Response
mRNA: messenger RNA
OS: overall survival
RPA: replication protein A
RPPA: reverse phase protein microarray
SCLC: small cell lung cancer
siCDC25C: small interfering CDC25C
siRNA: small interfering RNA
ssDNA: single‐stranded DNA
TMA: tissue microarray
WB: western blot

## 1 Introduction

Small cell lung cancer (SCLC) harbors very frequent mutations in p53 and Rb, which are key cell cycle regulators in normal cells [1, 2]. In the absence of p53 suppressor activity, SCLC cells mainly rely on the Ataxia Telangiectasia and Rad3 (ATR)‐Checkpoint Kinase 1 (Chk1) pathway to overcome replication stress in the event of DNA damage [3, 4]. Chk1 is a vital serine/threonine protein kinase that is responsible for cell cycle checkpoint‐mediated DNA damage response [5]. In the absence of cell cycle arrest, DNA damage will not be repaired, and cells will enter mitosis with damaged DNA that will lead to cancer cell death [6]. Studies showed that Chk1 also regulates the firing of dormant origins [4], which are initiation zones or clusters of DNA replication [7]. With impaired Chk1 function, cells can end up in replication catastrophe because of chromosome instability [8, 9]. Several reports indicate that combining Chk1 inhibition and chemotherapy agents such as platinum, gemcitabine, pemetrexed, doxorubicin or radiotherapy has additive antitumor activity in different cancer types [10, 11]. In p53‐intact normal or cancer cells, DNA damage can activate p53, which protects cells from premature cell cycle progression caused by Chk1 inhibition [12, 13]. Thompson *et al*. [14] showed that Chk1 inhibition enhances cisplatin cytotoxicity in cisplatin‐sensitive SCLC cell lines. Byers and colleagues have presented promising results using Chk1 inhibition alone or combined with cisplatin in SCLC xenograft models [15].

The process of cell cycle is rigorous and tightly controlled. The critical components of regulatory systems are the cyclin‐dependent kinase (CDK)/Cyclin complex. The rapid activation of the CDK1/Cyclin B complex allows the cells to undergo the transition from G2 to M phase, and this transition is usually inhibited by Wee1 kinase but promoted by Cdc25 phosphatases. Wee1 is a critical negative regulator of G2/M transition and belongs to the nuclear serine/threonine protein kinase family. The human ‘Wee’ protein kinase family consists mainly of Wee1 and Myt1. Wee1 mainly inhibits the activation of CDK1/Cyclin B complex by CDK1 phosphorylation (Tyr15), thus inhibiting cell entry into mitosis. In contrast to Cdc25 activity, Wee1 can phosphorylate CDK1 (Tyr15), thereby inhibiting the catalytic activity of CDK1 and block mitotic entry. Thus its activity likely impacts the sensitivity of cells to Chk1 inhibitor as well. High expression of Wee1 has been associated with poor survival and higher recurrence rate in melanoma, ovarian carcinoma, gastric cancer and glioblastoma [16]. The mechanisms of resistance to Chk1 inhibitors are largely unclear, and acquired resistance to Chk1 inhibitors has not been investigated so far. In a recent report, Teicher *et al*. screened 63 human SCLC lines and three NSCLC lines for response to 103 anticancer agents approved by the U.S. Food and Drug Administration and 423 investigational agents. Results showed that agents targeting nuclear kinases are effective in SCLC lines [17, 18]. Besides the activity of Chk1 inhibition in combination with chemotherapy, Chk1 inhibitor has also been shown to have antitumor effects as a single agent, by causing DNA double‐strand breaks in S phase and by promoting premature G2/M transition, leading to mitotic catastrophe [19]. However, only 15% of a large panel of cell lines of different tumor types were sensitive to Chk1 inhibitors; the majority of cell lines were primarily insensitive (intrinsic resistance). It is likely that many different mechanisms can lead to the resistance, such as failure to activate CDK2, even in the presence of Chk1 inhibition [20].

Here, we explored the potential contribution of Wee1 to acquired resistance to prexasertib and other Chk1 inhibitors in SCLC models.

## 2 Materials and methods

### 2.1 Cell lines

The human SCLC cell lines GLC4 (obtained from S. De Jong, Groningen University, Netherlands), H82, H128, H209, H792 and DMS114 (purchased from ATCC, Manassas, VA, USA) were cultured in RPMI 1640 medium Glutamax (Thermo Fisher, Waltham, MA, USA) supplemented with 10% fetal bovine serum and 1% penicillin‐streptomycin at 37 °C in a 5% CO_2_ incubator. Acquired prexasertib‐resistant cell lines were generated by continuous exposure to drug‐containing medium. The concentration of prexasertib was titrated up to 1 µm. Resistance levels were determined by cell viability assays. Mycoplasma testing was performed regularly using MycoAlert Detection Kit (Lonza, Walkersville, MD, USA).

### 2.2 Drugs

Cisplatin was purchased from Sigma (St. Louis, MO, USA). Prexasertib was provided by Eli Lilly or purchased from MedChemExpress (Monmouth Junction, NJ, USA). The prexasertib provided by Eli Lilly was used to establish acquired‐resistant cell lines, evaluate the characteristics of the resistant cell lines, do the inhibition experiments by Wee1 inhibitor and detect the downstream proteins. All other experiments were performed with prexasertib purchased from MedChemExpress. Several experiments were performed using both drugs with superimposable results. AZD7762, PF477736, RO3306, K3861, THZ1, BIRB796 and MK1775 were purchased from Selleckchem (Houston, TX, USA).

### 2.3 Cell viability assay

CellTiter‐Glo® (Promega, Madison, WI, USA) was used to measure cell viability. Cells were plated in 96‐wells and treated with drugs for 72 h. The signals were read by Glomax Multi‐detection system (Promega). The IC_50_ of each reagent was calculated by calcusyn software (BIOSOFT, Cambridge, UK).

### 2.4 Western blot analysis

Western blot (WB) was carried out using the SDS/PAGE system (Bio‐Rad, Philadelphia, PA, USA), including the precast gradient gel 4–20% and TurboTransfer system, according to the manufacturer’s instructions. The intensities of bands were detected using genetools software (SynGene, Frederick, MD, USA) and standardized by the intensity of α‐tubulin or β‐actin (both antibodies were from Sigma). All other antibodies used were purchased form Cell Signaling Technology (Danvers, MA, USA), with the exception of caspase‐2 (EMD Millipore, Burlington, MA, USA), phospho‐CDC25A(Ser76) (Abcam, Cambridge, MA, USA) and phospho‐CDK2(Tyr15) (Novus, Centennial, CO, USA).

### 2.5 Cell cycle analysis

Cells (3–6 × 10^5^) were seeded in 6‐well plates, washed with cold PBS and fixed with ice‐cold 75% ethanol in PBS. Propidium iodide was added, and DNA content was measured by FACStar plus (Becton Dickinson, Franklin Lakes, NJ, USA) and analyzed by the ModFit LT program.

### 2.6 siRNA knockdown and plasmid transfection

Cells were transfected with specific small interfering (si)RNA for Wee1, CDK1, CDC25C or scrambled siRNA (Dharmacon, Lafayette, CO, USA) using Lipofectamine RNAiMAX (Thermo Fisher) for 24 h, prior to treatment with designated drugs. A number of main experiments were also performed with a different pool of siRNA for Wee1 (Santa Cruz, Santa Cruz, CA, USA). The concentration of the siRNA used for transfection is 40 nm. The siRNA sequences are available in Table S1. The Wee1 plasmid was obtained from Origene (Rockville, MD, USA) and transfected using X‐tremeGENE DNA transfection reagent (Sigma) or the Cell Line Nucleofector Kit with Nucleofector Device (Lonza) according to the manufacturer’s protocol.

### 2.7 DNA copy number assay

DNA copy number variation (CNV) analysis was performed using TaqMan DNA copy number assay kit (Thermo Fisher) according to the manufacturer’s protocol. RPPH was used as the reference gene. Results were analysed by copy caller v2.1 (Roche, Mannheim, Germany).

### 2.8 qRT‐PCR

Total RNA was extracted using Allprep DNA/RNA mini kit (Qiagen, Germantown, MD, USA) and qRT‐PCR was performed using the QuantiTect SYBR Green RT‐PCR Kit (Qiagen). All procedures were performed on the ABI 7900HT Fast Real‐Time PCR System according to the manufacturer’s instructions. All primers data are described in Table S2.

### 2.9 Tissue microarray and immunohistochemistry

We used a tissue microarray (TMA) containing 157 cases of resected SCLC, described before [21]. The procedures for immunohistochemistry (IHC) have also been previously described [22]. All primary antibodies were obtained from Cell Signaling Technology. Two independent pathologists (B.K. and J.C.) reviewed the stained slides in a blinded fashion. The percentages of tumor cells with positive staining to Wee1 or Chk1 (from 0% to 100%) were used to calculate mean Wee1 and Chk1 expression scores. The median was used as the cutoff value for Chk1, and the optimal value obtained from the ROC curve was used as the cutoff value for Wee1.

### 2.10 Reverse phase protein microarray

The parental and resistant cell lines of H792 and GLC4 cells were collected before and after treatment with 100 nm prexasertib for 24 h, washed with cold PBS twice, and then placed on dry ice and later assayed by reverse phase protein microarray (RPPA); details of the method are described elsewhere [23]. Arrays were probed with a total of 159 antibodies, of which 122 were phospho‐specific proteins (listed in Table S3).

### 2.11 Statistical analysis

Statistical analyses were determined by *t*‐test or one‐way ANOVA using graphpad prism V5.0 (GraphPad Software, La Jolla, CA, USA). Data were expressed as mean ± SD. All *P*‐values were two‐sided and were considered statistically significant if below 0.05. The Combination Index (CI) between two drugs was calculated by calcusyn using the Chou–Talalay method [24], which defines additive effect (CI = 1), synergism (CI < 1) and antagonism (CI > 1).

## 3 Results

### 3.1 Establishment and characterization of SCLC cell lines with acquired resistance to prexasertib

**Fig. 1:**
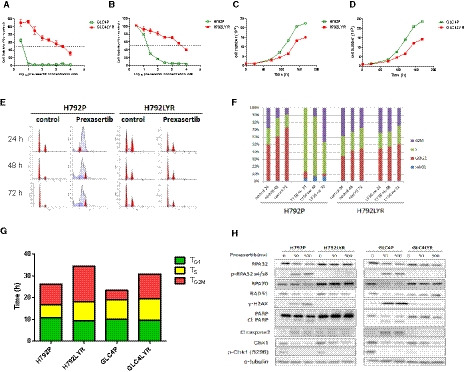
Characteristics of parental and prexasertib acquired‐resistant SCLC cell lines. Cell viability of parental and prexasertib‐resistant SCLC cell lines: (A) GLC4 and (B) H792. Cells were treated with different concentrations of prexasertib for 72 h and cell viabilities were detected by CellTilter‐Glo. (C) Cell doubling times for H792P and H792LYR cell lines. (D) Cell doubling time for GLC4P and GLC4LYR cell lines. Cells were seeded in six‐well plates, 103 per well, and counted every 24 h for 196 h. Data are represented as mean ± SD of three independent experiments. (E) Cell cycle analysis by PI staining and flow cytometry for H792P and H792LYR cells with or without 50 nm prexasertib treatment for 24, 48 and 72 h. (F) Quantification of the cell cycle analysis. (G) Histogram shows the different cell cycle phases: TG1, TS and TG2/M for H792, GLC4 parental and resistant cells, respectively. (H) Cells were treated with prexasertib 50 or 500 nm for 48 h, and apoptosis or DNA repair response proteins as indicated were detected by WB.

Two SCLC cell lines, NCI‐H792 (p53 mut, Rb wild‐type) and GLC4 (p53 mut, Rb mut), were exposed to increasing concentrations of prexasertib (from 100 nm to 1 µm) for over a period of at least 3 months. We obtained two acquired‐resistant SCLC cell lines, H792LYR and GLC4LYR, with IC_50_ for prexasertib of 6828 and 5200 nm, respectively, which are more than 100 and 500 times higher than those of the parental cells (50 nm for H792 and 10 nm for GLC4) (Fig. 1A,B). Compared with their parental cells, the acquired‐resistant cells had a longer doubling time: 34.3 (H792LYR) vs. 26.1 h (H792), and 30.7 (GLC4LYR) vs. 23.3 h (GLC4) (Fig. 1C,D). The prexasertib‐resistant cells were also resistant to other Chk1 inhibitors – PF477736 and AZD7762 (Fig. S1), indicating cross‐resistance. In the cell cycle analysis, the untreated parental cells had a smaller percentage of G2/M cells compared with the untreated resistant cells. As Chk1 inhibition leads to apoptosis in S phase [25, 26], prexasertib treatment caused significant cell cycle arrest in S phase in the parental cells, at the cost of significant reduction in G0/G1 cells, followed by a relative increase in the G2/M phase at later time points; in the resistant cells, there was no significant difference between untreated and treated cells and the percentage of cells in the different phases remains relatively stable (Fig. 1E,F). Applying Steel’s formula [27], in both H792 and GLC4 there was an increase of TG2M in the resistant cells compared with parental cells. For H792 parental cells, TG1, TS and TG2M were 10.7, 6.0 and 9.4 h, respectively. For H792‐resistant cells, TG1, TS and TG2M were 9.3, 8.7 and 16.3 h, respectively. Similarly, in the GLC4 parental cells, TG1, TS and TG2M were 10.0, 9.0 and 4.3 h, respectively. In the resistant cells, TG1, TS and TG2M were 9.7, 9.8 and 11.2 h, respectively. The extension of the doubling time in resistant cells was therefore mainly due to the prolongation of the G2/M phase (Fig. 1G).

### 3.2 Prexasertib‐induced DNA damage and apoptosis are reduced in resistant cells

Prexasertib can generate double‐stranded DNA breaks, leading to replication catastrophe. The abundance of single‐stranded (ss) DNA during replication stress exhausts the available pool of Replication Protein A (RPA; it protects ssDNA from nuclease), increasing the likelihood that unprotected ssDNA will be cleaved by endonucleases [28]. RPA is subsequently replaced by the DNA recombinase RAD51, which forms extended helical filaments on the ssDNA [29]. RPA32, RPA70 and RAD51 were decreased in the parental cells in a dosage‐dependent manner upon prexasertib treatment, but not in the resistant cells (Fig. 1H). It has been shown that ataxia telangiectasia mutated (ATM) regulates chromatin loading of ATM and Rad‐3‐related (ATR) under DNA damage [30], and ATR inhibits replication catastrophe by preventing exhaustion of RPA. We observed that both ATM and ATR kinases are activated in the resistant cells while prexasertib reduced the expression of ATM and ATR in parental cells (Fig. S2). Furthermore, cleaved PARP, cleaved caspase 2 and γH2AX were decreased in the resistant cells compared with the parental cells (Fig. 1H), indicating that Chk1 inhibition induces less DNA damage and apoptosis in the resistant cells.

### 3.3 Increased Wee1 expression contributes to acquired resistance to prexasertib

**Fig. 2:**
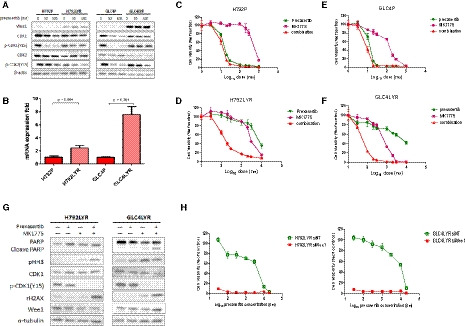
Wee1 expression is elevated in prexasertib‐resistant cells and contributes to acquired resistance. (A) H792 and GLC4 parental and resistant cells were exposed to prexasertib at the indicated concentrations for 48 h, and then, Wee1 and cell cycle regulated proteins as indicated were detected by WB. (B) Graphical depiction of Wee1 mRNA expression in H792 and GLC4 parental and resistant cells by qRT‐PCR. Data are presented as mean ± SD of three independent experiments. Unpaired and two‐tailed Student’s *t*‐test. Cell viability analysis for (C) H792P, (D) H792LYR, (E) GLC4P and (F) GLC4LYR cells. Cells were exposed to different concentrations of prexasertib, MK1775 or both for 72 h, and cell viability was detected by CellTiter‐Glo. (G) Cell cycle and DNA damage‐related proteins were detected by WB in H792LYR and GLC4LYR cells. Cells were exposed to prexasertib 50 nm or MK1775 1 µm or both as indicated for 48 h, and then the cell lysate was used in WB analysis. (H) Cell cytotoxicity analysis for siControl or Wee1 siRNA‐transfected H792LYR and GLC4LYR cells. Cells were transfected with siControl or siWee1 by RNAiMAX reagent. At 24 h after transfection, cell viability was detected by CellTiter‐Glo.

Because Wee1 is a known important negative regulator of G2/M transition and cell cycle checkpoint [31], we further investigated whether Wee1 might play a key role in the cell cycle progression of the resistant cells. The messenger (m)RNA and protein levels of Wee1 were significantly up‐regulated in H792LYR and GLC4LYR cells compared with the parental cells, irrespective of prexasertib exposure (Fig. 2A,B). Wee1 is a critical negative regulatory kinase, which phosphorylates and inactivates CDK1 to block G2/M transition. To determine whether Wee1 plays a causal role in the resistance to Chk1 inhibitors, we examined the cytotoxic effect of prexasertib in combination with the Wee1 inhibitor MK1775 in acquired‐resistant cell lines (H792LYR and GLC4LYR) and four additional parental SCLC cells (H82, H128, H209 and DMS114). The combination of prexasertib and MK1775 resulted in a synergistic effect in these cells (Fig. 2C,F and Fig. S3A–C); the addition of MK1775 drastically improved the sensitivity of the H792LYR and GLC4LYR cells to prexasertib (approximately 70‐fold from 8415 to 119 nm for H792LYR and 100‐fold from 4700 to 33 nm for GLC4LYR). H128 cells expressing more Wee1 showed better survival under prexasertib treatment but there was no significant correlation between RB expression and drug sensitivity. MK1775 alone and in combination with prexasertib caused reduction of phospho‐CDK1(Y15) levels in both prexasertib‐resistant cells. Accordingly, the mitotic marker phospho‐histone H3 (pHH3) increased when cells were exposed to the Wee1 inhibitor, and combination of MK1775 and prexasertib induced γH2AX and PARP cleavage (Fig. 2G). To confirm the specificity of Wee1 inhibition, we used a different siRNA to knockdown the Wee1 expression in resistant cells. The results were similar to those obtained with the Wee1 inhibitor MK1775. The IC_50_ for prexasertib decreased over 1000‐fold in the resistant cells when Wee1 was knocked down (Fig. 2H). WB results also demonstrated abrogation of G2 arrest (increased pHH3) and more DNA damage upon knockdown of Wee1 (Fig. S3D). These data suggest that Wee1 plays an important role in acquired resistance to Chk1 inhibition.

### 3.4 Wee1 is amplified in GLC4‐resistant cells

**Fig. 3:**
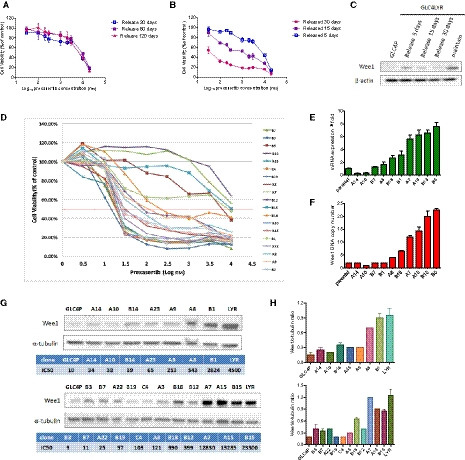
Wee1 amplification in GLC4‐resistant cells is highly correlated with the degree of resistance. Cell viability of H792LYR (A) or GLC4LYR cells (B) by CellTiter‐Glo. Cells were cultured without prexasertib for different time periods as indicated. Data are presented as mean ± SD of three independent experiments. (C) GLC4LYR cells were cultured without prexasertib for different time periods, Wee1 protein expression was detected by WB. (D) Cell cytotoxicity of prexasertib for 19 single clones of GLC4LYR by CellTiter‐Glo. (E) Wee1 mRNA expression of 10 GLC4LYR single clones, detected by qRT‐PCR. (F) Wee1 DNA copy number of 10 GLC4LYR single clones, analyzed by DNA copy number assay. (G) 18 GLC4LYR single clones in which Wee1 protein expression was detected by WB. (H) Graphical representation of the western blot (G) is shown in the bar graph where the band intensity of Wee1 is normalized to the α‐tubulin.

The acquired resistance was stable in H792LYR cells in the absence of prexasertib exposure for more than 4 months (Fig. 3A). In contrast, GLC4LYR became less resistant after culture in the absence of drug for 30 days, and the IC_50_ for prexasertib decreased from 4700 to 80 nm (Fig. 3B). Expression of Wee1 also decreased progressively following the removal of prexasertib in the culture of GLC4LYR (Fig. 3C). To determine whether the reduction of prexasertib resistance was due to clonal expansion of less‐resistant GLC4LYR cell populations, we cloned the cells that had been off drug for 30 days by the limited dilution method. Among the clones analyzed, there was a large variation in their sensitivity to prexasertib, which inversely correlated with the levels of Wee1 expression (rho = 0.927, *P* < 0.001, Spearman test) (Fig. 3D–H). Several clones retained Wee1 gene amplification and the number of gene copies correlated with the level of resistance to prexasertib (rho = 0.865, *P* = 0.001, Spearman test) (Table S4). These data indicate that Wee1 amplification does indeed contribute to the acquired resistance to Chk1 inhibitor in GLC4 cells.

Although we did not observe Wee1 amplification in H792LYR cells (Fig. S4A), its expression was significantly higher in H792LYR than in H792P cells (Fig. S4B,C). Interestingly, the expression of E2F1, which binds the promoter region of Wee1 to regulate Wee1 transcription [32], was also higher in H792LYR than in H792P cells (Fig. S4B,C). The siRNA knockdown of E2F1 in H792LYR cells caused a significant reduction of Wee1 expression and increased the sensitivity of the cells to prexasertib (Fig. S4D–F). Therefore, Wee1 may contribute to the resistance to Chk1 inhibitor via different mechanisms in different cells, i.e. by gene amplification or transcriptional up‐regulation.

**Fig. 4:**
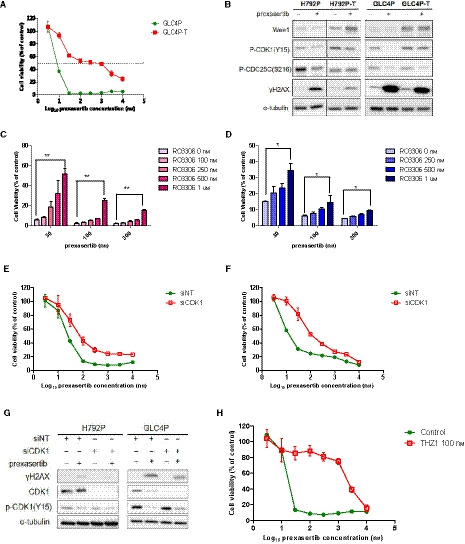
Resistance to Chk1 inhibitor is induced via CDK1 inhibition. (A) Cell viability of GLC4P and GLC4P cells transfected with Wee1 plasmid (GLC4P‐T); GLC4P‐T cells were transfected by electroporation and analyzed by CellTiter‐Glo. (B) Wee1 and cell cycle and DNA damage‐related proteins for H792 and GLC4 parental and transfected cells; parental cells were transfected by electroporation for 48 h and then exposed to prexasertib 50 nm for another 48 h. Protein expression was detected by WB. (C) GLC4P and (D) H792P cells were exposed to indicated concentrations of the CDK1 inhibitor RO3306 and prexasertib. Cell viabilities were observed by CellTiter‐Glo. (E) H792P and (F) GLC4P cells were transfected with CDK1 siRNA for 24 h and the cell viability was measured by CellTiter‐Glo. (G) CDK1 and DNA damage marker proteins for H792 and GLC4 parental cells transfected by control siRNA and CDK1 siRNA. The parental cells were transfected with control siRNA or CDK1 siRNA for 24 h and then exposed to prexasertib 50 nm for another 48 h. Protein expression was detected by WB. (H) Cell viability of GLC4P cells and cells exposed to the CDK7 inhibitor THZ1 100 nm for 72 h, assessed by CellTiter‐Glo. All data for cell viability test are presented as mean ± SD of three independent experiments. Unpaired and two‐tailed Student’s *t*‐test. *P < 0.05; **P < 0.01; ns, not significant.

To further validate the role of Wee1 in Chk1 inhibitor resistance, we examined the sensitivity of GLC4P cells after transfection with Wee1 expression vector by electroporation. Cell viability analysis and WB results indicate that the transfected cells become resistant to prexasertib compared with parental cells (Fig. 4A,B). Since the main function of Wee1 is to inhibit CDK1 through phosphorylation, we then asked whether Wee1‐mediated resistance to Chk1 inhibitor was regulated through inhibition of CDK1 activity. We observed that cells treated with the CDK1 inhibitor RO3306 or CDK1 siRNA were less sensitive to prexasertib (Fig. 4C–F). Compared with prexasertib alone, the combination of prexasertib and CDK1 siRNA reduced the DNA damage (Fig. 4G). Interestingly, we did not observe such DNA damage reduction when we used the CDK2 inhibitor K3861 (Fig. S5). Whereas Wee1 phosphorylates Y15 and T14 in CDK1 to inactivate its kinase activity, CDK7, a CDK‐activated kinase (CAK), activates CDK1 by phosphorylating CDK1 at T161 to promote G2/M transition [33]. In fact, treatment of GLC4 parental cells with the CDK7 inhibitor THZ1 also led to resistance to prexasertib (Fig. 4H). These data indicate that prexasertib sensitivity depends upon CDK1 activation, and Wee1‐mediated resistance to Chk1 inhibitor is a result of dysregulation of CDK1.

### 3.5 CDC25C plays a role in Chk1 resistance

**Fig. 5:**
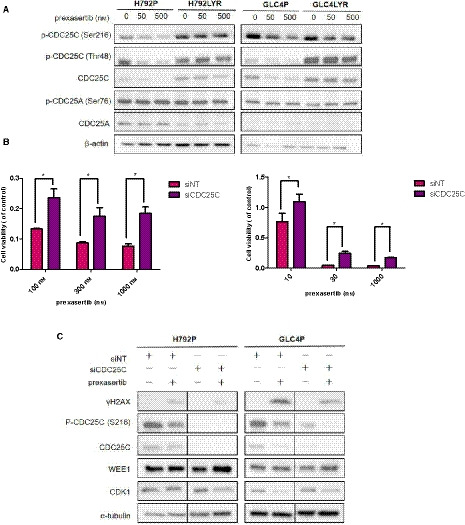
Inactivation of CDC25C contributes to the resistance to Chk1 inhibition. (A) CDC25 homolog family protein expression in H792 and GLC4 parental and resistant cells. The cells were exposed to indicated concentrations of prexasertib for 48 h and WB was performed. (B) Cell viabilities for GLC4P and H792P cells by CellTiter‐Glo. Cells were transfected with small interfering (si)CDC25C for 24 h to knock down CDC25C expression and then exposed to different concentrations of prexasertib for 72 h. *P < 0.05. (C) Expression of CDC25C and other proteins as indicated in H792P and GLC4P cells by WB. Alpha‐tubulin was used as a loading control. Cells were transfected with siCDC25C for 24 h, then exposed to prexasertib 50 nm for 48 h. All data for cell viability test are presented as mean ± SD of three independent experiments. Unpaired and two‐tailed Student’s *t*‐test. *P < 0.05; **P < 0.01; ns, not significant.

The G2/M transition is negatively regulated by Wee1 and promoted by CDC25. CDC25C activates CDK1 by dephosphorylating Y15 and T14 residues in CDK1 to promote cell cycle progression, whereas activated Chk1 induces CDC25C phosphorylation at the S216 site, thereby inhibiting its phosphatase activity [34]. Interestingly, the Chk1‐resistant cells H792LYR and GLC4LYR had much higher levels of phospho‐CDC25C(S216) (inactive form) compared with their parental cells (Fig. 5A). Furthermore, the levels of phospho‐CDC25C(Ser216) were significantly reduced in the parental H792 and GLC4 cells but remained high in the resistant cells after exposure to prexasertib (Fig. 5A). It is conceivable that CDK1 remains in an inactive state in the resistant cells due to the lack of CDC25C phosphatase activity, thus preventing the cells from entering mitosis even in the presence of Chk1 inhibitor. Therefore, we further asked whether CDC25C is required for cell death induced by Chk1 inhibition. Knockdown of CDC25C in H792P and GLC4P resulted in significant increase of cell viabilities when treated with prexasertib (Fig. 5B). CDC25C knockdown in parental cells also mitigated prexasertib‐induced DNA damage, evidenced by a reduction of γH2AX (Fig. 5C). These data suggest that inactivation of CDC25C may also contribute to the resistance to Chk1 inhibitor.

### 3.6 Reverse phase protein assay (RPPA) identifies P38MAPK as potential contributor to acquired resistance

**Fig. 6:**
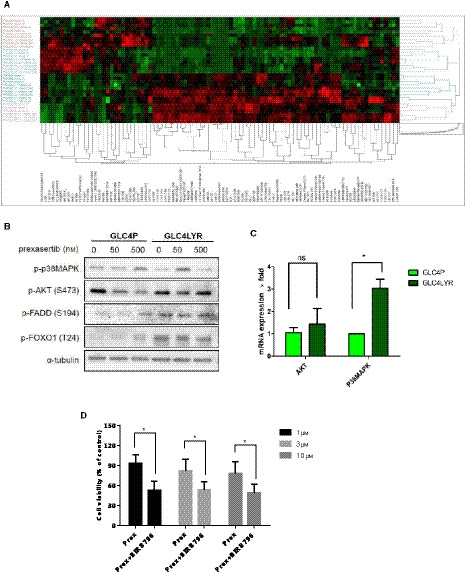
P38MAPK contributes to acquired resistance in GLC4LYR‐resistant cells. (A) RPPA results and heat map. The heat map shows 12 clusters, each of which is in triplicate, showing the relative protein expression levels and profiles in H792 and GLC4 parental and resistant cells at baseline and after exposure to 100 nm prexasertib for 2 and 24 h. (B) p‐P38MAPK(T180/Y182), p‐FADD(S194), p‐AKT(S473) and p‐FOXO1(T24) protein expression in GLC4 parental and resistant cells. The cells were exposed to indicated concentrations of prexasertib for 48 h and proteins were extracted and subjected to WB. (C) Graphical depiction of P38MAPK and AKT mRNA expression in GLC4 parental and resistant cells by qRT‐PCR. Data are presented as mean ± SD of three independent experiments. (D) Cell viability analysis for GLC4LYR‐resistant cells by CellTiter‐Glo. Cells were exposed to indicated concentrations of prexasertib with or without a fixed concentration of 3 µm of the P38MAPK inhibitor BIRB796 for 72 h. Data are presented as mean ± SD of three independent experiments. Unpaired and two‐tailed Student’s *t*‐test. *P < 0.05; **P < 0.01; ns, not significant.

To explore other potential contributors to resistance mechanisms, we used RPPA to perform a broader based pathway activation analysis and examine protein changes in H792 and GLC4 parental and resistant SCLC cell lines, exposed to prexasertib treatment for 24 h. Of importance is that the Wee1 was not included in the RPPA panel. The heat map (Fig. 6A) shows resistant cells compared with parental cells: the top 10 up‐regulated proteins in the two resistant cell lines are involved in cell cycle regulation, proliferation and apoptosis (Table S5). Also, we compared the protein changes induced by treatment and focused on the top 10 up‐regulated proteins in the resistant cell lines (Supporting Information Table S6). We verified the expression of four phosphorylated proteins by WB: phospho‐p38MAPK(T180/Y182), phospho‐Akt(S473), phospho‐FOXO1(T24) and phospho‐FADD(S194) (Fig. 6B). We were able to confirm that the expression of these four phosphorylated proteins was higher in GLC4LYR cells than in parental cells, and remained high after exposure to prexasertib. However, we were unable to confirm this finding in H792 cells (Supporting Information Fig. S6A). In some of those phosphorylated proteins, such as p‐FOXO1(T24), the baseline expression in parental and resistant H792 cell lines is much lower. Furthermore, we detected p38MAPK and AKT mRNA expression in GLC4 cell lines. Compared with parental cells, the p38MAPK mRNA expression was two times higher in GLC4‐resistant cells (*P* < 0.05) but AKT expression was not significantly different. No significant differences in expression of p38MAPK and AKT mRNA were observed in H792 cell lines (Figs 6C and S6B). Also, compared with parental cells, after prexasertib treatment, phosphorylated p38MAPK (p‐p38MAPK) and phosphorylated MK2 (p‐MK2) protein expression increased dramatically in resistant cells and p‐MK2 increased after exposure to prexasertib (data not shown). In addition, GLC4‐resistant cells became more sensitive to prexasertib and displayed more cell death when the p38MAPK inhibitor BIRB796 was added (Fig. 6D).

### 3.7 Higher Wee1 expression correlates with better prognosis and higher Chk1 expression in resected SCLC patients

To determine whether expression of Wee1 or Chk1 correlates with the prognosis of SCLC patients, we performed IHC staining of these two proteins using a TMA containing 149 resected SCLC specimens. Patients with higher Wee1 expression (> 50% of cancer cells) had a better overall survival (median OS: not reached for Wee1‐high group vs. 37.2 months for Wee1‐low patients, *P* = 0.038) (Supporting Information Fig. S7A). Furthermore, Wee1 expression positively correlated with Chk1 expression (rho = 0.610, *P* < 0.001 Spearman *t*‐test) (Fig. S7B) but there was no significant correlation between Chk1 expression and overall survival (Fig. S7C).

## 4 Discussion

Platinum‐based chemotherapy has been the cornerstone treatment for SCLC for decades, but unfortunately no major progress in chemotherapy has been made after the FDA approval of topotecan, a topoisomerase I inhibitor, 20 years ago. The development of new systemic treatments is crucial in this disease, which is often widely spread to distant sites at diagnosis. Chk1 is a key regulator of the cell cycle and plays a central role in normal DNA replication, resolving replication stress, mitosis entry and cytokinesis under DNA damage. Inhibition of Chk1 in the absence of DNA damage can cause impaired DNA replication, loss of DNA damage checkpoints, premature entry into mitosis with highly fragmented DNA, and cell death via replication catastrophe [19]. High expression of Chk1 is present in many solid tumors such as breast, ovarian, gastric and colorectal cancer, and is usually associated with poor prognosis. Since the ATR‐Chk1 pathway is crucial to overcome replication stress and for cell cycle arrest in the event of DNA damage, Chk1 is potentially an important therapeutic target in tumors, which frequently harbor mutated tumor suppressors such as p53 and Rb. The sensitivity of tumor cells to the inhibition of Chk1 has been correlated with basal levels of and/or induced DNA damage and replication stress.

Since most chemotherapeutic drugs induce DNA damage, the attractiveness of Chk1 inhibitors probably lies mainly in combination with the aim of enhancing DNA damage induced by chemotherapy or other drugs that are involved in the DNA damage response signal network, such as Wee1 inhibitors [35]. The development of Chk1 inhibitors has been burdened by toxicity and acquired resistance [36, 37]. More specific Chk1 inhibitors and the use of combinations may improve these issues; in particular, synergistic combinations with chemotherapeutic agents may theoretically allow decreasing the dosage for each single agent [38, 39]. Prexasertib is a second‐generation ATP‐competitive Chk1 inhibitor that is highly selective for the autophosphorylation at site S296. Prexasertib treatment displayed single agent activity in c‐myc overexpressing subsets of SCLC cell lines as well as in combination with cisplatin or the PARP inhibitor olaparib [40]. Prexasertib combined with PARP inhibitor also had a synergistic effect in gastric cancer [41]. At present, 14 clinical trials of prexasertib are ongoing or have been completed, most of which are phase I or phase II studies in colorectal cancer, triple negative breast cancer, head and neck cancer, and SCLC. Recently, prexasertib showed clinical activity and tolerability in patients with BRCA wild‐type high‐grade serous ovarian carcinoma [42].

As with most drugs, resistance is also a major problem in the development of Chk1 inhibitors. Only 15% of tumor cell lines responded to the Chk1 inhibitor MK8776 as a single agent, and primary resistance does not appear to be due to lack of drug bioavailability or defects in Chk1. Even the resistant cells still showed auto‐phosphorylation of ser296 after 24 h incubation with a topoisomerase inhibitor and cultured with Chk1 inhibitor for more than 7 days. The resistance could be overcome by inhibition of Wee1 [20]. Interestingly, in SCLC cell lines with primary resistance to the Wee1 inhibitor MK1775, AXL was overexpressed, which led to activation of the ERK/p90RSK and thus to recruitment of Chk1 as a parallel DNA damage repair pathway [43].

Our results indicate that Wee1 overexpression plays an important role in acquired resistance to the Chk1 inhibitor prexasertib and was due to increased Wee1 copy number or enhanced transcription. In the cell cycle progression, the G2/M transition depends on the activity of CDK1, which mainly relies on the balance between the activities of the Wee1 kinase and the CDC25C phosphatase, and Wee1 and CDC25 tightly control the molecular switch for this transition. Given this tight regulation of the DNA Damage Response (DDR), targeting multiple kinases in this signal pathway could result in a selective killing of tumor cells, especially in SCLC, which mostly harbors p53 and Rb mutations. Combining two or more DDR signal pathway inhibitors could be a valuable therapeutic strategy. Synergy has been achieved using Chk1 and Wee1 inhibitors together in melanoma, lymphoma, leukemia and other solid tumors [44, 45]. Our results indicate that the combination of prexasertib and the Wee1 inhibitor MK1775 was able to overcome drug resistance to Chk1 in SCLC cells, where single agents were ineffective.

Overexpression of Wee1 is expected to suppress the CDK1 activity, leading to cell cycle arrest at the G2 phase. We showed that the resistant cells have a longer doubling time due to G2 phase extension. SCLC patients with higher Wee1 tumor expression had a better prognosis compared with the patients with lower expression in a large cohort of resected SCLC patients in our study. Theoretically, cells with higher Wee1 expression would have a longer doubling time and this may translate in longer PFS and survival in SCLC patients, because their tumor grows more slowly. Yoshida *et al*. [46] reported similar results in NSCLC patients, where Wee1 expression was inversely correlated with Ki‐67 staining (proliferation).

CDC25C has an opposite effect on CDKs to Wee1. Our results indicate that CDC25C stays in an inactive status in resistant cells by maintaining significantly higher levels of phospho‐CDC25C (Ser216) in resistant cells compared with parental cells, even after treatment with prexasertib. The phosphorylation at Ser216 of CDC25C inhibits its phosphatase activity, thus preventing CDC25C from removing the inhibitory phosphate Y15 on CDK1, which is essentially an activator of cell cycle progression, and finally preventing cells from entering mitosis even in the presence of Chk1 inhibitor. Similar results have been reported in ovarian cancer cells [47].

We were aware that mechanisms for acquired resistance are multiple and we used a screening approach to investigate other potential mechanisms that could explain the Chk1 resistance. We used the RPPA analysis for this screening, and all top 10 up‐regulated proteins in resistant cells were involved in apoptosis, proliferation or cell cycle. Only two proteins, p38MAPK and TNF‐R1, in the top 10 up‐regulated proteins induced by exposure to prexasertib in resistant cells vs. parental cells could be seen in both cell lines. Our results suggest that p38MAPK may also play a role in acquired resistance to prexasertib. Besides the ATR/Chk1 and ATM/Chk2 signal pathways, it was reported that in the absence of p53, cells depend on a third cell cycle checkpoint pathway involving p38MAPK/MK2 for cell cycle arrest and survival after DNA damage. In the absence of MK2 in p53‐deficient cells, cytotoxic drug exposure may lead to elimination of S and G2/M phase checkpoint and mitotic catastrophe [48, 49]. Our results show that p38MAPK mRNA expression is much higher in resistant cells than in parental cells. The RPPA results demonstrated that the activated phospho‐p38MAPK in resistant cells increased significantly when exposed to prexasertib, which could not be observed in parental cells. The p38MAPK inhibitor BIRB796 was to some extent able to resensitize the Chk1 inhibitory activity in resistant cells.

TNF‐R1 was the top up‐regulated protein induced by exposure to prexasertib in resistant vs. parental cells in both cell lines. TNF‐1R is the receptor for TNF and is involved in inducing cell apoptosis via TRADD and caspases. Further studies are warranted to confirm these findings and to investigate further the potential mechanism underlying this overexpression.

## 5 Conclusions

Our study suggests that Wee1 up‐regulation is a major and novel mechanism of acquired resistance to Chk1 inhibitors in SCLC. Combination of Chk1 and Wee1 inhibitors may overcome the resistance. Combination studies of Chk1 inhibitors such as prexasertib and other agents may lead to synergistic interactions. Combinations with immune checkpoint inhibitors may be of particular interest, given the recent positive results of atezolizumab in combination with platinum chemotherapy in SCLC patients with extensive disease [50].

## Conflict of interest

EP and MP are inventors of US government and university‐assigned patents and patent applications that cover aspects of the technologies discussed such as Reverse Phase Protein Microarrays. As inventors, they are entitled to receive royalties as provided by US law and George Mason University policy. EP and MP receive royalties from Avant Diagnostics. LL and EP are consultants for and shareholders in Avant Diagnostics, Inc.; EP is a consultant for and shareholder in Perthera, Inc. All authors declare no potential conflicts of interest.

## Author contributions

XZ, Y‐WZ, CW and GG designed the research. XZ, I‐KK, BK, JJC, EI, JNM, SU and VC performed the research. MP and EP performed RPPA analysis. XZ, I‐KK and GG wrote the paper.

## Supporting information


**Fig. S1.** Cell viabilities for H792 and GLC4 parental and resistant cells for PF477736, AZD7762 and cisplatin.
**Fig. S2.** ATR and ATM protein expression in H792P and H792LYR cells by WB.
**Fig. S3.** Wee1 inhibition increases lethality to Chk1 inhibition in SCLC.
**Fig. S4.** Wee1 contributes to the acquired resistance to Chk1 inhibitor through E2F1.
**Fig. S5.** Cell viability of H792P cells under Chk1 or Cdk2 inhibition.
**Fig. S6.** Protein expression of p‐P38MAPK, p‐FADD, p‐AKT and p‐FOXO1 (A), and AKT and P38MAPK mRNA levels in H792 parental and resistant cells.
**Fig. S7.** High Wee1 expression correlates with better prognosis in SCLC patients.
**Table S1.** Sequences of the siRNA used.
**Table S2.** Primers used for the qRT‐PCR.
**Table S3.** Antibodies included in the RPPA assay.
**Table S4.** IC_50_ for prexasertib, Wee1 mRNA expression level and Wee1 DNA copy number for different GLC4LYR single clones.
**Table S5.** Expression of the top 10 up‐regulated proteins in H792 and GLC4 resistant cells vs. parental cells.
**Table S6.** Expression of the top 10 up‐regulated proteins induced by exposure to prexasertib in parental and resistant cells.

## Data Availability

RPPA raw data can be obtained from the following link: http://capmm.gmu.edu/data
