# Supplementary material for: Acquired small cell lung cancer resistance to Chk1 inhibitors involves Wee1 up‐regulation
Source: Mol Oncol. 2021 Jan 26;15(4):1130–45. doi: 10.1002/1878-0261.12882 (PMC8024728; doi:10.1002/1878-0261.12882)
Supplement: Supplementary file 1 — Fig. S1. Cell viabilities for H792 and GLC4 parental and resistant cells for PF477736, AZD7762 and cisplatin. Fig. S2. ATR and ATM protein expression in H792P and H792LYR cells by WB. Fig. S3. Wee1 inhibition increases lethality to Chk1 inhibition in SCLC. Fig. S4. Wee1 contributes to the acquired resistance to Chk1 inhibitor through E2F1. Fig. S5. Cell viability of H792P cells under Chk1 or Cdk2 inhibition. Fig. S6. Protein expression of p‐P38MAPK, p‐FADD, p‐AKT and p‐FOXO1 (A), and AKT and P38MAPK mRNA levels in H792 parental and resistant cells. Fig. S7. High Wee1 expression correlates with better prognosis in SCLC patients. Table S1. Sequences of the siRNA used. Table S2. Primers used for the qRT‐PCR. Table S3. Antibodies included in the RPPA assay. Table S4. IC50 for prexasertib, Wee1 mRNA expression level and Wee1 DNA copy number for different GLC4LYR single clones. Table S5. Expression of the top 10 up‐regulated proteins in H792 and GLC4 resistant cells vs. parental cells. Table S6. Expression of the top 10 up‐regulated proteins induced by exposure to prexasertib in parental and resistant cells. [file MOL2-15-1130-s001.docx]

**Supplementary Figures**

**Figure S1. Cell viabilities for H792 and GLC4 parental and resistant cells for PF477736, AZD7762 and cisplatin.** Cells were exposed to indicated drugs at different concentration for 72 h, and then cell viabilities were detected by Cell Titer Glo.


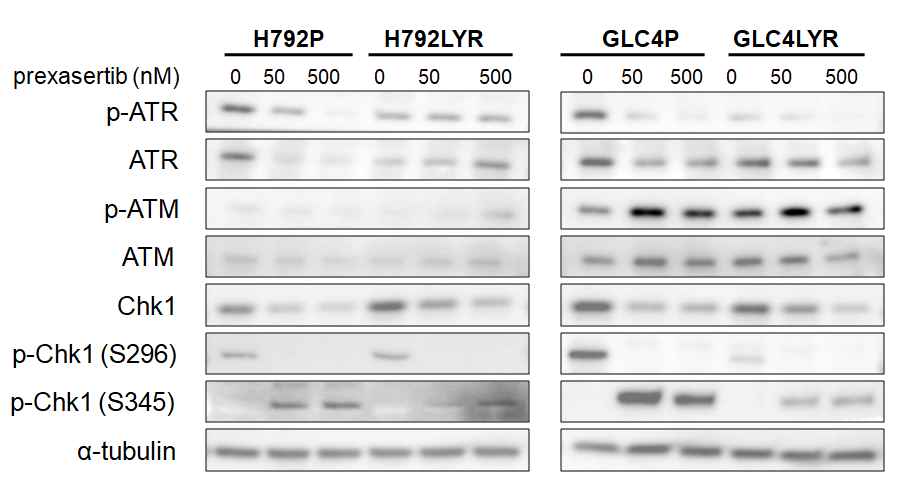

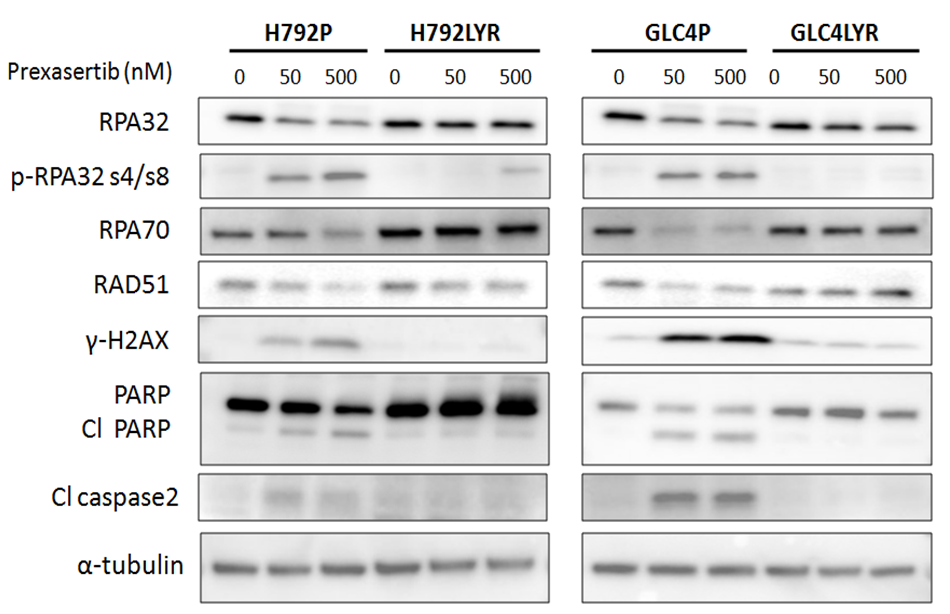


**Figure S2. ATR and ATM protein expression in H792P and H792LYR cells by WB.**

**Figure S3. Wee1 inhibition increases lethality to Chk1 inhibition in SCLC.** (A) The synergistic effect of prexasertib and Wee1 inhibitor MK1775 (CI<1) was detected in the H792 resistant cells. Cells were exposed to different concentrations of prexasertib and MK1775 as indicated for 72 hours, and then cell viabilities were detected by Cell Titer Glo. The synergistic effect was calculated by Calcusyn software using the Talalay-Chou method. (B) Cell viability in SCLC cells exposed to indicated inhibitor for 72 hours, measured by cell Titer Glo. (C) WB of RB and Wee1 in 4 SCLC cell lines. (D) Wee1, DNA damage, and cell cycle related proteins were detected by WB in GLC4LYR cells. Cells were transfected with siControl or siWee1 for 24 hours, and then exposed to prexasertib 50 nM or MK1775 1 μM or both, as indicated, for 48 hours.

**A B**

**
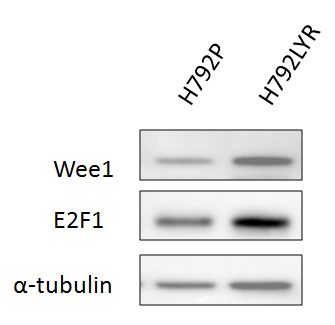
 C D**


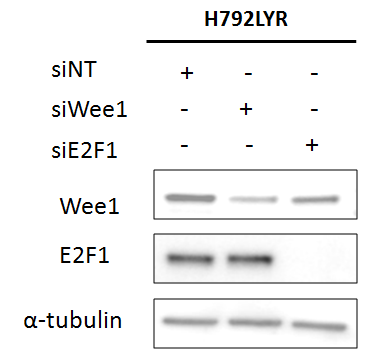


**E F**

**Figure S4. Wee1 contributes to the acquired resistance to Chk1 inhibitor through E2F1.** (A) Wee1 DNA copy number in H792P, H792LYR cultured in the medium with prexasertib (1 μM) and cultured with medium without prexasertib for 20 days. (B) Wee1 and E2F1 protein expression in H792P and H792LYR cells by WB. (C) mRNA expression level of Wee1 and E2F1 in H792P and H792LYR cells by qRT-PCR. (D) Wee1 and E2F1 expression in H792LYR cells. Cells were transfected with siWee1 and siE2F1 as indicated for 24 hours, and the target proteins were detected by WB. α-tubulin was used as a loading control. (E) mRNA expression level of Wee1 and E2F1 in H792LYR cells, by qRT-PCR. The cells were treated as panel C. (F) Cell viability in H792LYR cells transfected with siE2F1 for 24 hours then exposed to different concentrations of prexasertib as indicated, for 72 hours, detected by cell Titer Glo. ** means p<0.01.

**Figure S5. Cell viability of H792P cells under chk1 or cdk2 inhibition.** Cells were seeded in 96 wells plate and exposed to indicated concentrations of the CDK2 inhibitor K3861, prexasertib or the combination, for 72 hours, followed by cell viability assay by Cell-Titer Glo.


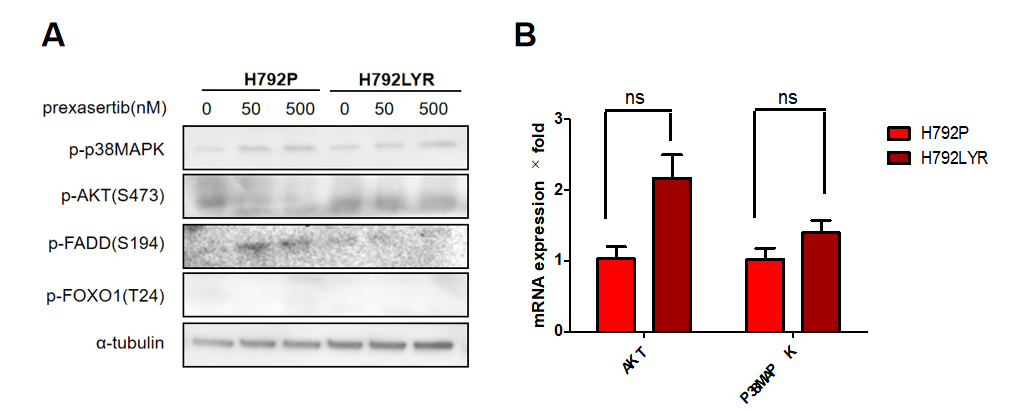


**Figure S6. Protein expression of p-P38MAPK, p-FADD, p-AKT and p-FOXO1 (A), and AKT and P38MAPK mRNA levels in H792 parental and resistant cells.** n.s = no significant difference.

A


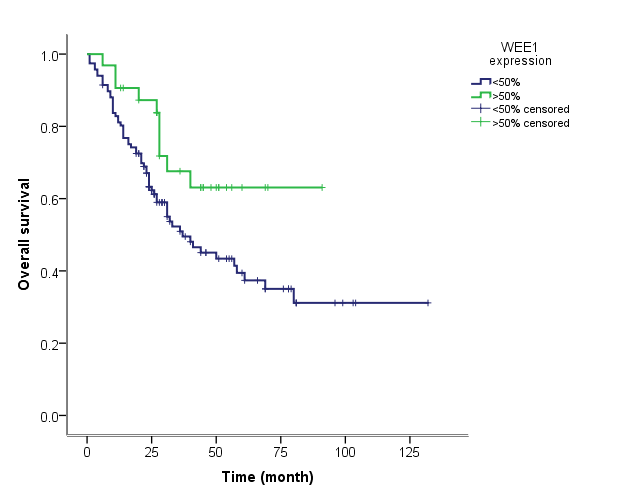


B

**C**


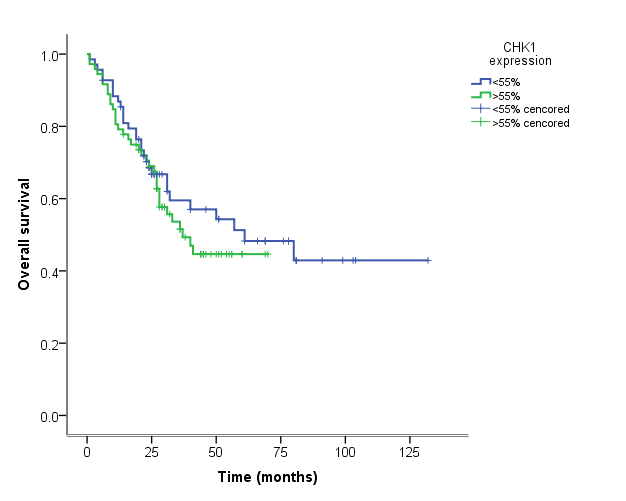


**Figure S7. High Wee1 expression correlates with better prognosis in SCLC patients.** (A) Compared to the SCLC patients with low Wee1 expression (≤50% positive cells), Resected SCLC patients with higher Wee1 expression (Wee1 staining positive percentage >50%) correlated with better overall survival (median OS: not reached, vs 37.2 months; log rank test p=0.038). (B) Wee1 expression was a positively correlated with Chk1 expression in resected SCLC patients (rho=0.610, p<0.001 Spearman test). (C) Resected SCLC patients with higher Chk1 expression (>55%) had a numerically longer survival than patients with lower Chk1 expression (<55%) but this difference was not statistically significant (median OS=61 months for lower expression patients v.s 37 months for higher expression patients, log rank= 0.642, p=0.423 K-M analysis).

**Supplementary Tables**

**Table S1. Sequences of the siRNAs used.**

**Wee1**

ON-TARGET SMARTpool siRNA; target sequences

5) AAUAGAACAUCACGACUUA

6) AAUAUGAAGUCCCGGUAUA

7) GAUCAUAUGCUUAUACAGA

8) CGACAGACUCCUCAAGUGA

**CDK1**

ON-TARGET SMARTpool siRNA; target sequences

13)   GGUUAUAUCUCAUCUUUGA

14)   UCGGGAAAUUUCUCUAUUA

15)   GUAUAAGGGUAGACACAAA

16)   CAAACGAAUUUCUCGCAAA

**CDC25C**

ON-TARGET SMARTpool siRNA; target sequences

9)      GAAACUUGGUGGACAGUGA

10)   AGGAAGGGCUUAUGUUUAA

11)   GAGAGAGACACUUCCUUUA

12)   GGGCAAAUUUCUUGGUGAU

**Table S2. Primers used for the qRT-PCR**

| **Wee1** | |
| --- | --- |
| Forward | 5’-TGGAGATCAATGGCATGAAA-3’ |
| Reverse | 5’-AGTGCCATTGCTGAAGGTCT-3’ |
| **GAPDH** | |
| Forward | 5’-GAGTCAACGGATTTGGTCGT-3’ |
| Reverse | 5’-TTGATTTTGGAGGGATCTCG-3’ |
| **E2F1** | |
| Forward | 5’-ATGTTTTCCTGTGCCCTGAG-3’ |
| Reverse | 5’-ATCTGTGGTGAGGGATGAGG-3’ |
| **β-actin** | |
| Forward | 5’-GGACTTCGAGCAAGAGATGG-3’ |
| Reverse | 5’-AGCATGTGTTGGCGTACAG-3’ |
| **P38MAPK** | |
| Forward | 5’-TGCACATGCCTACTTTGCTC-3’ |
| Reverse | 5’-AGGTCAGGCTTTTCCACTCA-3’ |
| **AKT** | |
| Forward | 5’-ACAAGGACGGGCACATTAAG-3’ |
| Reverse | 5’-GTCATTGTCCTCCAGCACCT-3’ |
| **MK2** | |
| Forward | 5’-AACGCCATCCTGAAACTCAC-3’ |
| Reverse | 5’-TCTCTGGACCCAGCACTTCT-3’ |

**Table S3. Antibodies included in the RPPA assay.**

| **Antibody** | **Vendor** | **Catalogue Number** |
| --- | --- | --- |
| Jak1 (Y1022/1023) | Cell Signaling | 3331 |
| Jak2 (Y1007) | Cell Signaling | 4406 |
| Smad2 (S245/250/255) | Cell Signaling | 3104 |
| Smad2 (S465/467) | Cell Signaling | 3101 |
| Stat3 (Y705) (D3A7) | Cell Signaling | 9145 |
| Stat5 (Y694) | Cell Signaling | 9351 |
| Stat6 (Y641) | Cell Signaling | 9361 |
| TGF-Beta (56E4) | Cell Signaling | 3709 |
| TNF-R1 (C25C1) | Cell Signaling | 3736 |
| PD-L1 (E1L3N) XP | Cell Signaling | 13684 |
| eIF4E (S209) | Cell Signaling | 9741 |
| eIF4G (S1108) | Cell Signaling | 2441 |
| eNOS/NOS III (S116) | Upstate | 07-357 |
| FAK (Y576/577) | Cell Signaling | 3281 |
| FKHR (S256) | Cell Signaling | 9461 |
| FoxO1 T24/FoxO3a T32 | Cell Signaling | 9464 |
| LKB1 (S334) | Cell Signaling | 3055 |
| mTOR (S2448) | Cell Signaling | 2971 |
| 4E-BP1 (S65) | Cell Signaling | 9451 |
| Raf (S259) | Cell Signaling | 9421 |
| A-Raf (S299) | Cell Signaling | 4431 |
| B-Raf (S445) | Cell Signaling | 2696 |
| c-Abl (T735) | Cell Signaling | 2864 |
| c-Abl (Y245) | Cell Signaling | 2861 |
| c-Raf (S338) (56A6) | Cell Signaling | 9427 |
| CREB (S133) | Cell Signaling | 9191 |
| EGFR (Y1068) | Cell Signaling | 2234 |
| EGFR (Y1148) | BioSource | 44-792 |
| EGFR (Y1173) | BioSource | 44-794 |
| Elk-1 (S383) | Cell Signaling | 9181 |
| ErbB2/HER2 (Y1248) | Imgenex | IMG-90189 |
| ErbB3/HER3 (Y1289) (21D3) | Cell Signaling | 4791 |
| ERK (T202/Y204) | Cell Signaling | 9101 |
| GSK-3a/B (S21/9) | Cell Signaling | 9331 |
| GSK-3beta (S9) | Cell Signaling | 9336 |
| IGF-1 Rec (Y1131)/Insulin Rec (Y1146) | Cell Signaling | 3021 |
| IGF-1R (Y1135/36)/IR (Y1150/51) (19H7) | Cell Signaling | 3024 |
| IRS-1 (S612) | Cell Signaling | 2386 |
| MEK1/2 (S217/221) | Cell Signaling | 9121 |
| PTEN (S380) | Cell Signaling | 9551 |
| p27 (T187) | Zymed | 71-7700 |
| p38 MAPK (T180/Y182) | Cell Signaling | 9211 |
| p53 (S15) | Cell Signaling | 9284 |
| p70 S6 Kinase (S371) | Cell Signaling | 9208 |
| p70 S6 Kinase (T389) | Cell Signaling | 9205 |
| p90RSK (S380) | Cell Signaling | 9341 |
| Shc (Y317) | Upstate | 07-206 |
| Src (Y527) | Cell Signaling | 2105 |
| Src Family (Y416) | Cell Signaling | 2101 |
| S6 Ribosomal Protein (S235/236) (2F9) | Cell Signaling | 4856 |
| S6 Ribosomal Protein (S240/244) | Cell Signaling | 2215 |
| SAPK/JNK (T183/Y185) | Cell Signaling | 9251 |
| PRAS40 (T246) | BioSource | 44-1100 |
| Acetyl-CoA Carboxylase (S79) | Cell Signaling | 3661 |
| Androgen Rec (S650) | Abcam | ab47563 |
| Androgen Rec (S81) | Millipore | 07-1375 |
| ASK1 (S83) | Cell Signaling | 3761 |
| Met (Y1234/1235) | Cell Signaling | 3126 |
| NF-kappaB p65 (S536) | Cell Signaling | 3031 |
| PDK1 (S241) | Cell Signaling | 3061 |
| ATP-Citrate Lyase (S454) | Cell Signaling | 4331 |
| BAD (S112) | Cell Signaling | 9291 |
| Beclin 1 | Cell Signaling | 3738 |
| Axl (Y702) | Cell Signaling | 5724 |
| Caspase-3, cleaved (D175) | Cell Signaling | 9661 |
| Caspase-7, cleaved (D198) | Cell Signaling | 9491 |
| Caspase-9, cleaved (D330) | Cell Signaling | 9501 |
| c-Kit (Y719) | Cell Signaling | 3391 |
| Chk-1 (S345) | Cell Signaling | 2341 |
| Chk-2 (S33/35) | Cell Signaling | 2665 |
| cPLA2 (S505) | Cell Signaling | 2831 |
| CrkL (Y207) | Cell Signaling | 3181 |
| Etk (Y40) | Cell Signaling | 3211 |
| Ezrin (Y353) | Cell Signaling | 3144 |
| FADD (S194) | Cell Signaling | 2781 |
| Histone H3 (S28) | Upstate | 07-145 |
| Lck (Y505) | Biosource | 44-850 |
| LIMK1 (T508)/LIMK2 (T505) | Cell Signaling | 3841 |
| PAK1 (S199/204)/PAK2 (S192/197) | Cell Signaling | 2605 |
| Paxillin (Y118) | Cell Signaling | 2541 |
| PDGF Receptor alpha (Y754) (23B2) | Cell Signaling | 2992 |
| SEK1/MKK4 (S80) | Cell Signaling | 9155 |
| PLK1 | Cell Signaling | 4535 |
| Histone H3 (S10) Mitosis Marker | Upstate | 06-570 |
| PKA C (T197) | Cell Signaling | 4781 |
| PKC alpha (S657) | Upstate | 06-822 |
| PKC delta (T505) | Cell Signaling | 9374 |
| Akt1/PKB alpha (S473) (SK703) | Upstate | 05-736 |
| PKC zeta/lambda (T410/403) | Cell Signaling | 9378 |
| Ret (Y905) | Cell Signaling | 3221 |
| Ron (Y1353) | Epitomics | 5176-1 |
| RSK3 (T356/S360) | Cell Signaling | 9348 |
| 4E-BP1 (T70) | Cell Signaling | 9455 |
| AMPKalpha (T172) (D79.5E) | Cell Signaling | 4188 |
| AMPKBeta1 (S108) | Cell Signaling | 4181 |
| ATF-2 (T69/71) | Cell Signaling | 9225 |
| ATF-2 (T71) | Cell Signaling | 9221 |
| ATR (S428) | Cell Signaling | 2853 |
| Aurora A (T288)/B (T232)/C (T198) (D13A11) | Cell Signaling | 2914 |
| Caveolin-1 (Y14) (EPR2288Y) | Epitomics | 2267-1 |
| Cofilin (S3) (77G2) | Cell Signaling | 3313 |
| eIF2alpha (S51) (119A11) | Cell Signaling | 3597 |
| eNOS (S113) | Cell Signaling | 9575 |
| FKHRL1/FOX03 (S253) | Upstate | 06-953 |
| FOXM1 (T600) | Cell Signaling | 14655 |
| HSP90a (T5/7) | Cell Signaling | 3488 |
| MDM2 (S166) | Cell Signaling | 3521 |
| MSK1 (S360) | Cell Signaling | 9594 |
| p70 S6 Kinase (T412) | Upstate | 07-018 |
| PAK1 (T423)/PAK2 (T402) | Cell Signaling | 2601 |
| PAK2 (S20) | Cell Signaling | 2607 |
| PARP, cleaved (D214) | Cell Signaling | 9541 |
| Pyk2 (Y402) | Cell Signaling | 3291 |
| Rb (S780) | Cell Signaling | 3590 |
| PKC theta (T538) | Cell Signaling | 9377 |
| GSK-3alpha (S21) (46H12) | Cell Signaling | 9337 |
| YAP (S127) (D9W2I) | Cell Signaling | 13008 |
| Bad (S136) | Cell Signaling | 9295 |
| Bad (S155) | Cell Signaling | 9297 |
| Bcl-2 (S70) (5H2) | Cell Signaling | 2827 |
| Bcr (Y177) | Cell Signaling | 3901 |
| Caspase-6, cleaved (D162) | Cell Signaling | 9761 |
| Catenin (beta) (S33/37/T41) | Cell Signaling | 9561 |
| Catenin (beta) (T41/S45) | Cell Signaling | 9565 |
| CrkII (Y221) | Cell Signaling | 3491 |
| eNOS (S1177) | Cell Signaling | 9571 |
| LKB1 (S428) | Cell Signaling | 3051 |
| MEK1 (S298) | Cell Signaling | 9128 |
| Mst1 (T183)/Mst2 (T180) | Cell Signaling | 3681 |
| PLC-gamma-1 | Cell Signaling | 2822 |
| Ras-GRF1 (S916) | Cell Signaling | 3321 |
| Vav3 (Y173) | Biosource | 44-488 |
| Akt | Cell Signaling | 9272 |
| Akt2 (5B5) | Cell Signaling | 2964 |
| Atg5 (part of Autophagy Ab Sampler #4445) | Cell Signaling | 2630 |
| Bad | Cell Signaling | 9292 |
| Bak | Cell Signaling | 3814 |
| Bax | Cell Signaling | 2772 |
| Bcl-2 | Cell Signaling | 2872 |
| Bcl-xL | Cell Signaling | 2762 |
| BIM | Cell Signaling | 2933 |
| CDK2 (78B2) | Cell Signaling | 2546 |
| c-Myc | Cell Signaling | 9402 |
| Biliverdin Reductase (BVR) | Stressgen | OSA-400 |
| Cu/Zn Superoxide Dismutase (SOD) | Stressgen | SOD-100 |
| DEPTOR | Millipore | 09-463 |
| ERK 1/2 | Cell Signaling | 9102 |
| Heme-Oxygenase-1 | Stressgen | SPA-894 |
| LC3B | Cell Signaling | 2775 |
| LDHA | Cell Signaling | 2012 |
| Mn Superoxide Dismutase (SOD) | Assay Design | SOD-110 |
| p62/SQSTM1 (D5E2) | Cell Signaling | 8025 |
| PI3-Kinase p110gamma | Cell Signaling | 4252 |
| Proteasome 20S C2 | Abcam | ab3325 |
| PTEN | Cell Signaling | 9552 |
| PUMA | Cell Signaling | 4976 |
| ST6GALNAC5 | Aviva | ARP49986 |
| VHL | Cell Signaling | 2738 |
| Vimentin | Cell Signaling | 3295 |

**Table S4. IC50s for prexasertib, Wee1 mRNA expression level and Wee1 DNA copy number for different GLC4LYR single clones**

| **Clone Number** | **IC50(nM)** | **mRNA(fold)** | **Copy number** |
| --- | --- | --- | --- |
| Parental | 18 | 1 | 2 |
| A14 | 9 | 0.33 | 2 |
| A10 | 11 | 0.37 | 0.86 |
| B7 | 15 | 1.27 | 2 |
| B1 | 55 | 3.12 | 2 |
| A8 | 26 | 1.69 | 4 |
| B18 | 678 | 3.16 | 6.5 |
| A7 | 10947 | 5.61 | 12 |
| A15 | 14163 | 6.27 | 14.25 |
| B15 | 13300 | 4.92 | 20 |
| B5 | 9045 | 7.53 | 22.5 |

**Table S5. Expression of the top 10 up-regulated proteins in H792 and GLC4 resistant cells compared to parental cells.**

| **H792** | | **GLC4** | |
| --- | --- | --- | --- |
| **Protein code** | **fold** | **Protein code** | **fold** |
| GSK-3beta S9 | 2.59 | PARP Cleaved D214 | 4.92 |
| PRAS40 T246 | 2.37 | HSP90 T5/7 | 2.68 |
| CrkL Y207 | 2.05 | p70S6 Kinase T389 | 2.22 |
| p70S6 Kinase S371 | 1.96 | FoxO1 T24/FoxO3a T32 | 2.18 |
| BAD S112 | 1.89 | S6 Ribosomal Protein S235/236 | 2.17 |
| eIF2alpha S51 | 1.88 | PDK1 S241 | 2.16 |
| Bad S136 | 1.77 | NF-kappaB P65 S536 | 1.96 |
| MEK 1/2 S217/221 | 1.75 | PKC theta T538 | 1.94 |
| PARP Cleaved D214 | 1.54 | GSK-3beta S9 | 1.84 |
| GSK-3a/B S21/9 | 1.47 | Akt1/PKB alpha S473 | 1.84 |

**Table S6. Expression of the top 10 up-regulated proteins induced by exposure to prexasertib between parental and resistant cells**

| **H792** | | **GLC4** | |
| --- | --- | --- | --- |
| **Protein** | **Variation*** | **Protein** | **Variation*** |
| TNF-R1 | 3.29 | TNF-R1 | 3.62 |
| Src family Y416 | 2.95 | p38MAPK T180/Y182 | 2.41 |
| ERK 1/2 T202/Y204 | 2.53 | Smad2 S465/467 | 1.95 |
| AR S81 | 2.38 | Heme-Oxygenase-1 | 1.81 |
| eNOS/NOS III S116 | 2.17 | Stat6 Y641 | 1.57 |
| Bcr Y177 | 2.17 | Vimentin | 1.37 |
| p38MAPK T180/Y182 | 2.13 | PI3-Kinase p110gamma | 1.35 |
| Pyk2 Y402 | 2.05 | MDM2 S166 | 1.21 |
| eNOS S113 | 2.00 | Proteasome 20S C2 | 1.19 |
| DEPTOR | 1.67 | AR S650 | 1.18 |

* Variation = resistant cells (after exposed/baseline) - parental cells (after exposed/baseline)
